# Coenzyme Q10 Protects Hair Cells against Aminoglycoside

**DOI:** 10.1371/journal.pone.0108280

**Published:** 2014-09-29

**Authors:** Kazuma Sugahara, Yoshinobu Hirose, Takefumi Mikuriya, Makoto Hashimoto, Eiju Kanagawa, Hirotaka Hara, Hiroaki Shimogori, Hiroshi Yamashita

**Affiliations:** Department of Otolaryngology, Yamaguchi University Graduate School of Medicine, Ube, Yamaguchi, Japan; Universitat Pompeu Fabra, Spain

## Abstract

It is well known that the production of free radicals is associated with sensory cell death induced by an aminoglycoside. Many researchers have reported that antioxidant reagents protect sensory cells in the inner ear, and coenzyme Q10 (CoQ10) is an antioxidant that is consumed as a health food in many countries. The purpose of this study was to investigate the role of CoQ10 in mammalian vestibular hair cell death induced by aminoglycoside. Cultured utricles of CBA/CaN mice were divided into three groups (control group, neomycin group, and neomycin + CoQ10 group). In the neomycin group, utricles were cultured with neomycin (1 mM) to induce hair cell death. In the neomycin + CoQ10 group, utricles were cultured with neomycin and water-soluble CoQ10 (30–0.3 µM). Twenty-four hours after exposure to neomycin, the cultured tissues were fixed, and vestibular hair cells were labeled using an anti-calmodulin antibody. Significantly more hair cells survived in the neomycin + CoQ10 group than in the neomycin group. These data indicate that CoQ10 protects sensory hair cells against neomycin-induced death in the mammalian vestibular epithelium; therefore, CoQ10 may be useful as a protective drug in the inner ear.

## Introduction

Sensory hair cells are easily damaged by chemicals such as aminoglycosides, infection, and ischemia [1]. After hair cells are damaged, auditory and vestibular dysfunction is permanent; therefore, it is important to prevent the loss of hair cells of patients with inner ear diseases. Previous studies indicated that hair cell death was related to oxidative stress. Aminoglycosides are well-known ototoxic agents, and their ototoxicity is mediated by the generation of free radicals [2].

Recently, coenzyme Q10 (CoQ10) has attracted a great deal of public attention as a nutritional supplement; it is used world-wide for health promotion and anti-aging as an anti-oxidant agent. However, CoQ10 is extremely lipid-soluble and not easily absorbed by the body. Recently, water-soluble CoQ10 was developed to improve absorption of CoQ10 in the body [3]. Therefore, in the present study, we investigated the protective effect of water-soluble CoQ10 against hair cell degeneration induced by neomycin.

## Materials and Methods

### Animal Use and Care

CBA/N mice obtained from Kyushu Animal Company (Kumamoto, Japan) were used in this study. All mice (4–6 weeks old) were male and had normal Preyer’s reflexes. The experimental protocol was reviewed and approved by the Committee for Ethics on Animal Experiments at the Yamaguchi University School of Medicine. Experiments were conducted in accordance with these guidelines, Japanese federal law (No. 105), and Notification No. 6 of the Japanese government.

### Organ Culture of Utricles and Induction of Hair Cell Death

All animals were deeply anesthetized with an overdose of pentobarbital and immediately decapitated. The temporal bones were quickly removed and the individual vestibular organs were dissected in basal Eagle medium (Invitrogen, Carlsbad, CA) supplemented with Earle’s balanced salt solution (Invitrogen) (2∶1, v/v). Isolated utricles were moved into the culture medium, which consisted of basal Eagle medium supplemented with Earle’s balanced salt solution (2∶1, v/v) and 5% fetal bovine serum (Invitrogen). The free-floating utricles were incubated in 24-well tissue culture plates for 12 or 24 h at 37°C in a 5% CO_2_ and 95% air environment. To induce hair cell death, neomycin solution (10 mg/mL; Sigma, St. Louis, MO) was added into the culture wells to a final concentration of 1.0 mM. After the culture protocols were completed, the utricles were fixed with 4% paraformaldehyde (PFA) for 1 h at room temperature. Otoconia were gently removed from fixed utricles by a stream of phosphate buffered saline (PBS) applied via a 28 G needle and syringe. After rinsing with PBS, the samples were used in the assays outlined below.

### Preparation of coenzyme Q10 solution

Water soluble CoQ10 (Eisai Co., Tokyo, Japan) was used in this study and dissolved in the medium before initiation of culture.

### Immunohistochemistry for hair cell labeling

Fixed utricles were incubated in blocking solution (1% bovine serum albumin, 0.4% normal goat serum, 0.4% normal horse serum, and 0.4% Triton X-100 in PBS) overnight at 4°C. To label hair cells, a monoclonal antibody against calmodulin (Sigma) and a polyclonal antibody against calbindin (Chemicon, Temecula, CA) were used. Samples were incubated overnight at 4°C in the primary antibody solution (calmodulin 1∶150 or calbindin 1∶250 in the blocking solution). After washing with the blocking solution, the specimens were incubated in secondary antibodies diluted in blocking solution as follows: biotinylated horse anti-mouse IgG (1∶100; Vector Laboratories, Burlingame, CA) or Alexa 488-conjugated goat anti-mouse IgG (1∶500; Molecular Probes, Eugene, OR) in addition to Alexa 594-conjugated goat anti-rabbit IgG (1∶500; Molecular Probes). After rinsing with blocking solution, the utricles were mounted in Vectashield (Vector Laboratories) and coverslipped.

### Immunohistochemistry for production of 4-HNE

To evaluate the production of reactive oxygen species, 4-hydroxy-2-nonenal (4-HNE) production was investigated. The samples were fixed with 4% PFA after dissection. Next, utricles were incubated in a 1∶100 dilution of anti-4-HNE mouse monoclonal antibody (OXIS International, Inc., Portland, OR) overnight in a refrigerator (4°C). After the rinsing in the blocking solution, the samples were incubated with Alexa 488-conjugated goat anti-mouse IgG and Texas red-conjugated phalloidin (1∶100, Sigma) for 4 hours at room temperature. The fluorescence intensity of the immunohistochemistry was evaluated with the image analysis software: ImageJ. Six samples were used for the experiment. The average of the fluorescence intensity derived from utricles cultured with normal medium was defined as 1. The intensities in the other groups were shown by the relative value.

### Evaluation of the number of residual sensory hair cells

Utricles were examined under a fluorescence microscope (XF-EHD2, Nikon, Tokyo, Japan) to evaluate the survival of hair cells. Calbindin-positive and calmodulin-positive cells were counted as hair cells in the striolar region and extrastriolar region, respectively. The labeled hair cells were counted in two squares, 20 µm on a side, which were determined randomly in each utricle. Eight striolar and eight extrastriolar hair cell counts were averaged to produce one striolar and one extrastriolar hair cell density for each utricle examined. At least six utricles were examined for each experimental condition. All data were expressed in mean ± standard error. Data were analyzed with StatView version 5.0J for Macintosh (SAS institute Inc, Cary, NC). These hair cell dinsities were compared with Mann-Whitney’s U test to determine significant values. A level of P<0.05 was accepted as statistically significant.

## Results

### Effect of coenzyme Q10 on hair cell survival

**Figure 1:**
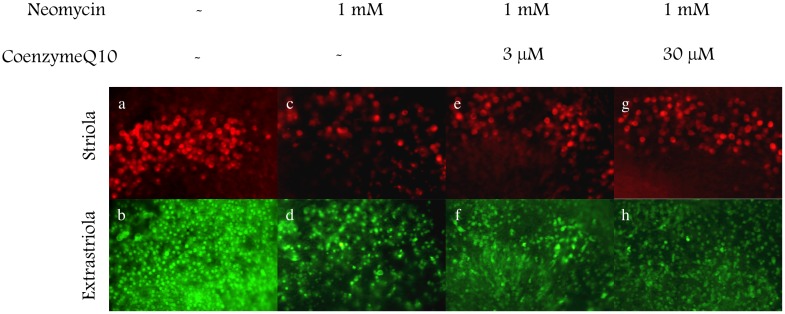
Protective effect of coenzyme Q10 against neomycin-induced cell death. Utricles were cultured for 24 h without neomycin (a, b), with neomycin (c, d) or with both neomycin and coenzyme Q10 (e–h). Death of striolar hair cells (red: calbindin) and extrastriolar hair cells (green: calmodulin) was inhibited by coenzyme Q10.

**Figure 2:**
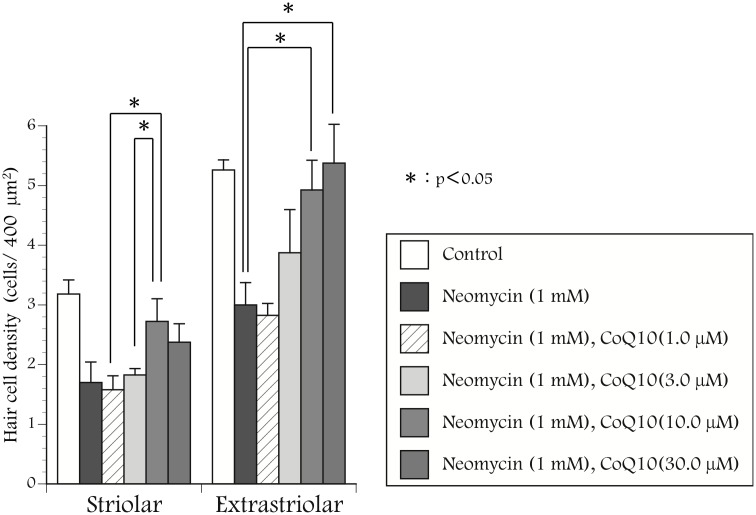
Relationship between the concentration of coenzyme Q10 and neomycin. The density of surviving hair cells was significantly higher in utricles treated with 10 µM and 30 µM of coenzyme Q10 and neomycin than in those treated with only 1 mM of neomycin. The protective effect of coenzyme Q10 was shown to be concentration-dependent; *: p<0.05.

To evaluate the effect of CoQ10 on the survival of hair cells treated with neomycin, utricles were cultured with neomycin (1 mM) and CoQ10 (1–30 µM) for 24 hours. The utricles were incubated for 2 hours with or without CoQ10 before exposure to neomycin. Calmodulin and calbindin were immunolabeled to detect residual hair cells (Fig. 1). In the medium with neomycin, the density of hair cells was reduced after 24 hours. More hair cells survived in the medium with both neomycin and CoQ10 than in the medium with neomycin alone. The density of hair cells in the cultured utricles is shown in Fig. 2. and Table 1. CoQ10 significantly suppressed the reduction of hair cell density induced by neomycin at concentrations of 10 and 30 µM.

**Table 1:** The density of surviving hair cells in the utricles cultured with CoQ10 and neomycin.

	Striolar (cells/400 µm^2^)	Extrastriolar (cells/400 µm^2^)
Control	3.18±0.24	5.26±0.17
Neomycin (1 mM)	1.70±0.34	3.00±0.38
Neomycin (1 mM), CoQ10 (1.0 µM)	1.58±1.23	2.83±0.20
Neomycin (1 mM), CoQ10 (3.0 µM)	1.83±0.11	3.88±0.72
Neomycin (1 mM), CoQ10 (10.0 µM)	2.73±0.38	4.93±0.50
Neomycin (1 mM), CoQ10 (30.0 µM)	2.38±0.31	5.38±0.65

### Coenzyme Q10 suppresses the production of 4-HNE

**Figure 3:**
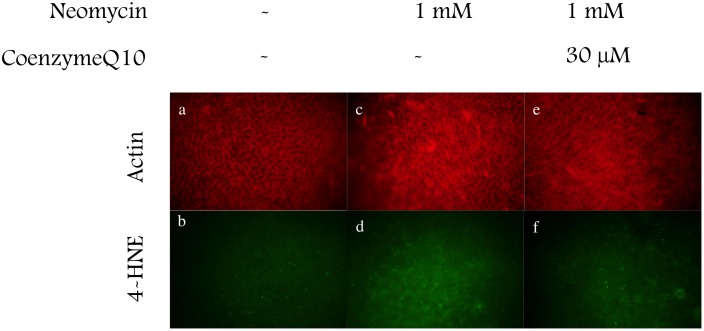
Inhibition of the production of 4-HNE in hair cells. Utricles were cultured for 12 h without neomycin (a, b), with neomycin (c, d), or with both neomycin and coenzyme Q10 (e, f). The hair cell layer was identified using Texas Red-conjugated phalloidin (red). 4-HNE was labeled using a specific antibody (green). Signals of 4-HNE were seen in utricle hair cells 12 h after exposure to neomycin. These 4-HNE signals were strongly inhibited by coenzyme Q10 treatment.

**Figure 4:**
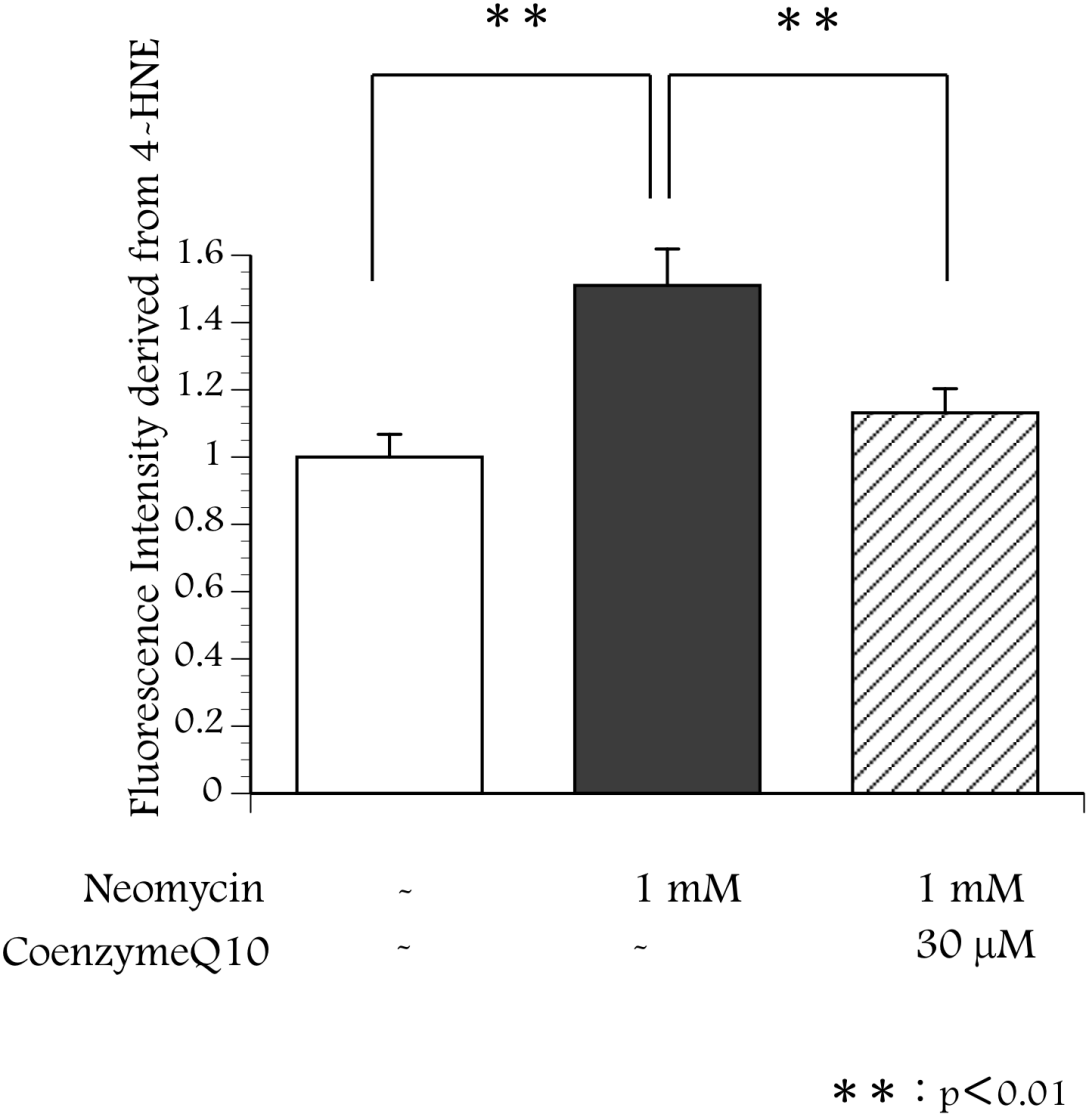
Fluorescence Intensity derived from 4-HNE. Fluorescence intensity of the immunohistochemistry was evaluated with the image analysis software: ImageJ. The fluorescence intensity derived from 4-HNE was significantly stronger in the utricles cultured with neomycin than without neomycin. The existance of coenzyme Q10 can inhibit the fluorescence intensity.

To detect the production of hydroxy radicals, immunohistochemistry was performed using an antibody against 4-HNE, which is the metabolic product of hydroxy radicals. Six cultured utricles were divided into three groups. Two utricles were cultured in the conventional medium described above for 14 hours. Two utricles were cultured in the conventional medium for 2 hours, and followed by culture for 12 hours after addition of neomycin (1 mM) into the medium. The other two utricles were cultured in medium containing neomycin (1 mM) and CoQ10 (30 µM) for 12 hours following culture in the normal medium (2 h). ß-actin was labeled with phalloidin conjugated with Texas Red to indicate the hair cell layer, and the fluorescence microscope was focused on the hair cell layer. Hair cells containing 4-HNE were not seen in utricles cultured for 12 hours without neomycin (Fig. 3b). Many hair cells containing 4-HNE appeared in utricles cultured with 1 mM neomycin (Fig. 3d). The 4-HNE signal was decreased in utricles cultured with neomycin and CoQ10 for 12 hours (Fig. 3f). These results indicate that CoQ10 suppressed the production of hydroxy radicals by utricles exposed to neomycin. The evaluation of the fluorescence intensity of the immunohistochemistry was shown in Fig. 4. The fluorescence intensity derived from 4-HNE was significantly stronger in the utricles cultured with neomycin than without neomycin. The existance of coenzyme Q10 can inhibited the fluorescence intensity.

## Discussion

Reactive oxygen species play an important role in hair cell death induced by aminoglycosides [4]. Many researchers have reported a relationship between the production of reactive oxygen species and hair cell damage induced by aminoglycosides [5]. Aminoglycosides are a class of compounds that are well known as specific ototoxic agents [6], and recent research suggests that hair cell death induced by these chemicals is closely related to apoptosis [7]. Therefore, many types of antioxidants are used to inhibit hair cell death induced by aminoglycosides, and antioxidant molecules are a candidate for the treatment of patients suffering from aminoglycoside-induced hearing loss and vestibular dysfunction [8].

In this study, we showed that the anti-oxidative agent CoQ10 suppresses hair cell death induced by neomycin. To elucidate the mechanism underlying the antioxidant activity of CoQ10, we assessed the production of 4-HNE, which is the metabolite of the hydroxy radical. In hair cells exposed to aminoglycoside, strong 4-HNE signals were observed, and CoQ10 inhibited this production of 4-HNE remarkably. This scavenging of free radicals is thought to have protected the hair cells from cell death.

Several studies have reported the effect of CoQ10 on inner ear function. Sato reported that CoQ10 was effective in promoting recovery from damage to auditory hairs caused by hypoxia [9]. Sergi reported an antioxidant function of idebenone (synthetic analogue of CoQ10) in protection from noise-induced hearing loss [10]. Recently, we reported that oral administration of CoQ10 suppressed inner ear damage after intense noise exposure [2]. In the present study, the direct activity of CoQ10 was determined using utricle organ cultures. CoQ10 has already been used for clinical trials; oral administration slowed the progressive deterioration of function in Parkinson’s disease [11], and CoQ10 prevented progressive hearing loss and improved blood lactate levels after exercise in patients with maternally inherited diabetes mellitus and deafness [12]. In these studies, CoQ10 was not associated with major side effects; therefore, it can safely be used for inner ear diseases.

We used a water-soluble form of CoQ10, which has traditionally been known as a lipid-soluble molecule and thus has been difficult to dissolve in medium. In addition, bioavailability of CoQ10 was low after oral administration. Recently, water-soluble CoQ10 was developed to improve absorption of CoQ10. Water-soluble CoQ10 showed higher uptake when administered in a fasting state or with food compared to lipid-soluble CoQ10 in a rat and human study [3], [13]. Fentoni et al. has reported the protective effect of another water-soluble CoQ10 in vivo experiments [14]. The water-soluble type of CoQ10 can be the tool for the treatment for inner ear diseases with hair cell damage.

## Conclusion

In the present study performed using cultured mice utricles, water-soluble CoQ10 protected sensory hair cells against neomycin-induced death in the mammalian vestibular epithelium. These results show that CoQ10 may be useful as a protective drug in the inner ear.
